# Age and metabolic syndrome are associated with unsatisfactory improvement in nocturia after holmium laser enucleation of the prostate

**DOI:** 10.3389/fsurg.2022.1063649

**Published:** 2023-01-10

**Authors:** Kaikai Lv, Yangyang Wu, Shuai Huang, Zhenjun Luo, Wenhui Lai, Qingyang Meng, Xinze Xia, Chao Lv, Xiaowei Hao, Tao Song, Qing Yuan

**Affiliations:** ^1^ Department of Urology, the Third Medical Centre, Chinese People's Liberation Army (PLA) General Hospital, Beijing, China; ^2^ Medical School of Chinese People's Liberation Army (PLA), Beijing, China; ^3^ Department of Postgraduate, Hebei North University, Zhangjiakou, Hebei, China; ^4^ Affiliated Hospital of Weifang Medical University, School of Clinical Medicine, Weifang Medical University, Weifang, China; ^5^ Department of Urology, Shanxi Medical University, Taiyuan, Shanxi, China

**Keywords:** benign prostate hyperplasia, lower urinary tract symptoms, nocturia, metabolic syndrome, holmium laser enucleation of the prostate

## Abstract

**Objective:**

To investigate the association between age, metabolic syndrome (MetS) and improvement in nocturia in patients with benign prostate hyperplasia (BPH) receiving holmium laser enucleation of the prostate (HoLEP).

**Methods:**

The retrospective study was conducted on patients treated for BPH using HoLEP between January 2021 and May 2022. Lower urinary tract symptoms (LUTS) were measured before surgery and at 3 months postoperatively using the International Prostate Symptom Score (IPSS). The criteria of the Adult Treatment Panel III (ATP III) were adopted to diagnose the MetS. Unsatisfactory improvement in nocturia was defined as <50% reduction in nocturia from baseline on the IPSS.

**Results:**

One hundred and seventy-five patients were eventually enrolled, with a median age of 69 years (IQR: 63/73). Unsatisfactory improvement in nocturia was reported in 95 patients (54%) after HoLEP. These patients were older (73; IQR: 67/79 vs. 66; IQR: 60/71, *P* < 0.001) and more likely to present with higher postoperative total (6; IQR: 4/9 vs. 3; IQR:2/5, *P* < 0.001), voiding (1; IQR: 0/3 vs. 1; IQR: 0/2, *P* = 0.017), and storage (4; IQR: 3/6 vs. 2; IQR: 1/4, *P* < 0.001) IPSS when compared to patients with satisfactory improvement in nocturia. Overall, 63 of 175 (36%) patients were diagnosed with MetS and of these, 44 (70%) reported unsatisfactory improvement in nocturia (*P* = 0.002) after HoLEP. Multivariate analysis revealed that age (OR = 1.117, 95% CI: 1.068–1.169, *P* < 0.001) and MetS (OR = 3.613, 95% CI: 1.727–7.562, *P* = 0.001) were independent risk factors for unsatisfactory improvement in nocturia after HoLEP.

**Conclusion:**

Our findings suggest that increased age and MetS were associated with unsatisfactory improvement in nocturia in patients with BPH after HoLEP. Lifestyle management, including weight loss, may be of great importance in the improvement of nocturia.

## Introduction

Nocturia, defined as waking up at night once or more to empty the bladder, is one of the most troublesome and treatment-resistant lower urinary tract symptoms (LUTS) secondary to benign prostate hyperplasia (BPH) (1, 2). It has been shown that more frequent nocturnal episodes are associated with poorer quality of life. Among men aged >70 years, 29%–59.3% had two or more nocturnal episodes, and were strongly associated with BPH (3). More than two episodes of nocturia are considered to have negative physical and mental effects, such as falling at night, fractures, depression and inefficiency (4).

To complicate matters further, nocturia is a common symptom of complex systemic diseases, including diabetes, hypertension, congestive heart failure and sleep apnea (5). Medical management of nocturia is less effective than surgical treatment in achieving significant improvement (1). However, it has been shown that surgical treatments, such as transurethral resection of the prostate (TURP), are also inadequate for reducing the frequency of BPH-related nocturia (6). Risk stratification of nocturia improvement after surgical treatment would benefit standardized management and remains to be further investigated.

Age is believed to be an independent risk factor for nocturia in both men and women, although the underlying pathological mechanisms are incompletely understood (7). Metabolic syndrome (MetS), a complex disorder with increasing worldwide prevalence, has been described as a combination of several metabolic abnormalities, including central obesity, hypertension, insulin resistance and dyslipidemia (8). Notwithstanding their inconsistent findings, several studies have conformed the association of components of metabolic syndrome (obesity, hypertension and diabetes) (9, 10) and lifestyle (smoking, alcohol intake and physical activity) (5) with nocturia.

Holmium laser enucleation of the prostate (HoLEP) has become one of the most important procedures in the treatment of BPH. However, postoperative storage symptoms, especially nocturia, still often show unsatisfactory improvement. Hence, it is important to predict the postoperative improvement in nocturia to better counsel patients preoperatively. To our knowledge, no works have been conducted to evaluate the role of metabolic factors on the likelihood of nocturia improvement after HoLEP in Chinese patients. Therefore, we performed the study to evaluate the association between MetS, age and improvement in nocturia in patients with BPH after HoLEP.

## Materials and methods

### Study design and participants

Clinical data of patients with symptomatic BPH treated at our hospital were collected from January 2021 to May 2022. A total of 348 patients underwent HoLEP performed by a single surgeon, and 175 of the 348 (50%) patients were eventually included in the study. The Institutional Review Board of our institute approved this retrospective study, and informed consent was obtained from all patients, as this study was a *post-hoc* analysis of prospective data. All the patients met the indications for surgery. We excluded patients with a history of urethral surgery, bladder stone, bladder or prostate cancer, neurogenic bladder, recurrent urinary tract infection, and diuretic use.

Patient characteristics, such as age, body mass index (BMI), systolic and diastolic blood pressure (SBP/DBP), and waist circumference (WC), were collected. With patients standing, we measured the WC midway between the lowest rib and the iliac crest. Operative information including operative time and hemoglobin (Hb) level change, was also recorded. In addition, blood samples were drawn from patients for analysis of blood glucose, triglycerides (TG), high-density lipoprotein (HDL) cholesterol, and total prostate-specific antigen (PSA) after an overnight fast.

### LUTS/BPH assessment

Prostate volume (PV) was calculated using transrectal ultrasound, according to the ellipsoidal formula:

$$PV=\;\frac{\pi }{6}\times width\times height\times depth$$

We evaluated LUTS using the International Prostate Symptom Score (IPSS) questionnaire, which includes voiding and storage IPSS. Voids per night were assessed using IPSS item 7 (nocturia). Unsatisfactory improvement in nocturia was defined as <50% reduction in nocturnal episodes after HoLEP compared to baseline.

### Mets definition

In this study, we applied the modified National Cholesterol Education Program/Adult Treatment Panel III (NCE/ATP III) to define MetS (11) as the presence of at least three of the following: (1) waist circumference ≥90 cm; (2) triglyceride level ≥1.7 mmol/L or taking drugs for hypertriglyceridemia; (3) HDL-cholesterol level <1.03 mmol/L or taking drug for low HDL-cholesterol; (4) fasting glucose ≥5.6 mmol/L or taking drugs for hyperglycemia; and (5) SBP ≥130 mmHg or DBP ≥85 mmHg or previously diagnosed hypertension.

### Follow-up

All patients were asked to complete the IPSS questionnaire in the outpatient clinic after 3 months. Patients who could not attend the outpatient clinic for follow-up were contacted *via* telephone to complete the IPSS questionnaire.

### Statistical analysis

After evaluation, the data set showed a non-normal distribution. Differences between groups were compared using the Mann-Whitney test for continuous variables and Pearson's chi-square test for categorical variables. Risk factors of unsatisfactory improvement in nocturia (<50% reduction) after HoLEP were assessed using binary logistic regression. The statistically significant variables in the univariate analysis were included in the multivariate model. Multicollinearity occurs when two closely related variables (i.e., MetS and fasting blood glucose) appear in the same multivariable analysis model, leading to unreliable results. Considering the risk of multicollinearity, MetS components were excluded from the multivariate model.

Statistical analyses were performed with SPSS version 26.0. All *P* values were two-sided with *P* < 0.05 considered statistically significant.

## Results

**Figure 1:**
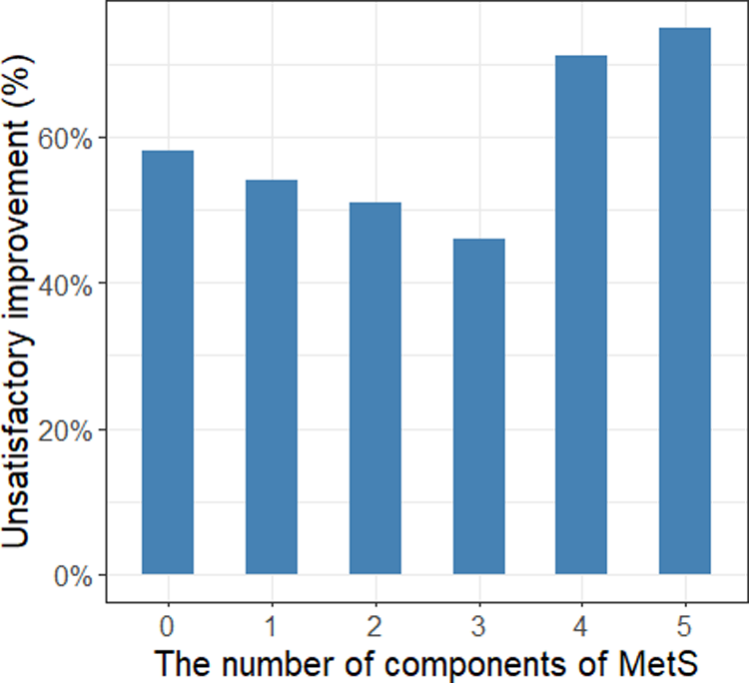
The association between the number of components of MetS and unsatisfactory improvement in nocturia.

The median age and BMI of the patients were 69 years and 24.2 kg/m^2^, respectively. Overall, 77 (44%) men smoked and 72 (41%) men had a history of alcohol consumption. The median prostate volume and median PSA of all patients were 62 cc and 3.37 ng/ml, respectively. The median number of voids per night was 4 before the operation and 2 after the operation. A total 95 of 175 (54%) patients showed unsatisfactory improvement in nocturnal episodes after HoLEP (Table 1). Sixty-three of 175 (36%) patients were diagnosed with MetS, and of these 44 (70%) reported unsatisfactory improvement in nocturia. Moreover, elevated blood pressure (73%), elevated waist circumference (62%) and elevated blood glucose (58%) were the three most reported components of MetS. In addition, the highest and lowest probabilities of unsatisfactory improvement in nocturia were observed in patients with five and three components of MetS, 75% and 46% respectively (Figure 1).

**Table 1:** Characteristics of the analyzed population (*n* = 175).

	Overall
Age, year	69 (63/76)
BMI, kg/m^2^	24.2 (22.3/26.2)
Smoking	77 (44%)
Alcohol	72 (41%)
Prostate volume, cc	62 (46/87)
Total PSA, ng/ml	3.37 (1.49/7.42)
Waist circumference ≥90 cm	109 (62%)
HDL <1.03 mmol/L or on drug treatment to reduced HDL-cholesterol	52 (30%)
Triglyceride ≥1.7 mmol/L or on drug treatment for elevated triglycerides	31 (18%)
Fasting glucose ≥5.6 mmol/L or on drug treatment for elevated glucose	58 (33%)
SBP ≥130 mmHg and/or DBP ≥85 mmHg or on antihypertensionsive drug treatment in a patient with history of hypertension	127 (73%)
MetS	63 (36%)
Preoperative IPSS	
Total	21 (17/27)
vIPSS	12 (10/16)
sIPSS	9 (7/12)
Nocturia	4 (3/5)
IPSS 3 mo	
Total	5 (3/7)
vIPSS	1 (0/3)
sIPSS	3 (2/5)
Nocturia	2 (1/3)
Nocturia improvement	80 (46%)

BMI, body mass index; PSA, prostate-specific antigen; HDL, high-density lipoprotein; SBP, systolic blood pressure; DBP, diastolic blood pressure; MetS, metabolic syndrome; IPSS, International Prostate Symptom Score; vIPSS, voiding International Prostate Symptom Score; sIPSS, storage International Prostate Symptom Score.

Overall, patients with unsatisfactory improvement in nocturia were older (73; IQR: 67/79 vs. 66; IQR: 60/71, *P* < 0.001) and more likely to suffer from diabetes (26/95: 27% vs. 12/80: 15%, *P* = 0.048) and MetS (44/95: 46% vs. 19/80: 24%, *P* = 0.002) than show improvement in nocturia. These patients presented higher level of fasting glucose (5.06; IQR: 4.61/5.96 vs. 4.76; IQR: 4.48/5.20, *P* = 0.001), more MetS components (2; IQR: 1/3 vs. 2; IQR:1/2, *P* = 0.011), higher postoperative total (6; IQR: 4/9 vs. 3; IQR: 2/5, *P* < 0.001), voiding (1; IQR: 0/3 vs. 1; IQR: 0/2, *P* = 0.017), storage (4; IQR: 3/6 vs. 2; IQR: 1/4, *P* < 0.001) IPSS (Table 2). However, there were no significant differences between the groups in terms of BMI, hypertension, SBP, DBP, smoking, alcohol intake, WC, TG, HDL, operation time, Hb change, prostate volume, total PSA level or preoperative IPSS.

**Table 2:** Patient's characteristics according to the improvement or not in nocturia after HoLEP.

	Satisfactory improvement	Unsatisfactory improvement	*P*
Patients	80	95	
Age, year	66 (60/71)	73 (67/79)	<0.001
BMI, kg/m^2^	24.3 (22.3/26.2)	24.2 (22.3/26.6)	0.690
Hypertension	33/80 (41%)	47/95 (49%)	0.277
SBP, mmHg	132 (125/137)	132 (126/140)	0.406
DBP, mmHg	76 (74/82)	76 (74/80)	0.930
Diabetes mellitus	12/80 (15%)	26/95 (27%)	0.048
Smoking	37/80 (46%)	40/95 (42%)	0.582
Alcohol	38/80 (48%)	34/95 (36%)	0.117
WC, cm	91 (87/96)	93 (87/97)	0.239
TG, mmol/L	1.00 (0.74/1.44)	1.09 (0.72/1.47)	0.654
HDL, mmol/L	1.21 (0.96/1.39)	1.17 (0.94/1.39)	0.509
Fasting glucose, mmol/L	4.76 (4.48/5.20)	5.06 (4.61/5.96)	0.001
Operation time, min	78 (60/100)	80 (60/100)	0.294
Hb change, g/L	9 (4/13)	7 (4/14)	0.724
MetS	19/80 (24%)	44/95 (46%)	0.002
No. MetS component	2 (1/2)	2 (1/3)	0.011
Prostate volume, cc	58 (43/82)	66 (47/89)	0.233
Total PSA, ng/ml	3.42 (1.57/8.72)	3.37 (1.40/6.90)	0.644
Preoperative IPSS			
Total	21 (17/27)	20 (16/27)	0.509
vIPSS	13 (10/16)	12 (10/15)	0.400
sIPSS	9 (7/12)	10 (6/12)	0.838
IPSS 3 mo			
Total	3 (2/5)	6 (4/9)	<0.001
vIPSS	1 (0/2)	1 (0/3)	0.017
sIPSS	2 (1/4)	4 (3/6)	<0.001

BMI, body mass index; SBP, systolic blood pressure; DBP, diastolic blood pressure; WC, waist circumference; TG, triglycerides; HDL, high-density lipoprotein; MetS, metabolic syndrome; PSA, prostatespecific antigen; IPSS, International Prostate Symptom Score; vIPSS, voiding International Prostate Symptom Score; sIPSS, storage International Prostate Symptom Score.

On crude logistic regression analysis, age (OR = 1.104; 95% CI: 1.058–1.151, *P* < 0.001), fasting glucose (OR = 1.468; 95% CI: 1.016–2.121, *P* = 0.041), and MetS (OR = 2.770; 95% CI: 1.440–5.327, *P* = 0.002) were predictors of unsatisfactory improvement in nocturia. Multivariate analysis revealed that age (OR = 1.117; 95% CI: 1.068–1.169, *P* < 0.001) and MetS (OR = 3.613; 95% CI: 1.727–7.562, *P* = 0.001) were independent risk factors for unsatisfactory improvement in nocturia after HoLEP (Table 3).

**Table 3:** Univariate and multivariate binary logistic regression to predict the risk of improvement in nocturia after HoLEP.

	Univariate odds ratio	*P*	Multivariate odds ratio	*P*
Age, year	1.104 (1.058–1.151)	<0.001	1.117 (1.068–1.169)	<0.001
BMI, kg/m^2^	1.029 (0.927–1.142)	0.588		
Hypertension	1.395 (0.765–2.541)	0.277		
DM	2.135 (0.997–4.573)	0.051		
Smoking	0.845 (0.464–1.539)	0.582		
Alcohol	0.616 (0.336–1.130)	0.118		
WC, cm	1.026 (0.983–1.070)	0.239		
TG, mmol/L	1.417 (0.641–3.132)	0.389		
HDL, mmol/L	0.867 (0.326–2.304)	0.775		
Fasting glucose, mmol/L	1.468 (1.016–2.121)	0.041		
MetS	2.770 (1.440–5.327)	0.002	3.613 (1.727–7.562)	0.001
PV, cc	1.005 (0.997–1.012)	0.228		
PSA, ng/ml	1.000 (0.976–1.024)	0.999		

BMI, body mass index; DM, diabetes mellitus; WC, waist circumference; TG, triglycerides; HDL, high-density lipoprotein; MetS, metabolic syndrome; PV, prostate volume; PSA, prostate-specific antigen.

## Discussion

This study evaluated the association between MetS, age and improvement in nocturia after HoLEP in Chinese patients with BPH. In our study, 95 (54%) patients reported unsatisfactory improvement in nocturia episodes after HoLEP, and of these 44 (46%) reported MetS. Results from our study showed that age and MetS were independent risk factors for unsatisfactory improvement in nocturia, as assessed by IPSS item 7, after HoLEP. Nocturia, the most frequently observed storage symptom, is still considered to be the most treatment-resistant LUTS among patients undergoing treatment for BPH (12). However, few studies have evaluated MetS and changes in nocturia in patients with BPH before and after surgery. Therefore, prospective studies are necessary to evaluate the impact of MetS and its components on the improvement of early long-term nocturia in patients with BPH.

Nocturia is an extremely common symptom that has been reported to be associated with a variety of comorbidities, including diabetes, obesity, coronary artery disease, depression and MetS (13). As people age, nocturnal episodes increases (5) and the prevalence of two or more voids per night can exceed 51% in men and 45% in women aged ≥60 years according to a 2019 study using the National Health and Nutrition Examination Survey (NHANS) (14). The potential causes are the loss of smooth muscle cells and the accumulation of collagen and fibrotic deposits in the aging bladder (15), leading to detrusor instability and deceased bladder capacity (16). When voiding ≥2 times per night, the elderly are at significantly increased risk of sleep disorders, falls, fractures, and daytime fatigue, leading to severe mortality (17).

Removal of bladder outlet obstruction promotes recovery of bladder function and alleviates associated symptoms such as nocturia. Wada et al. investigated the effects of TURP on nocturia and sleep disturbances in patients with LUTS/BPH. Their findings showed a significant improvement in nocturnal voids after surgical treatment, but nocturia showed the least improvement among items of the IPSS (18). Studies have reported a better nocturia response after TURP in patients with BPH compared with medications such as alpha-blockers, anticholinergics and desmopressin (19). Although TURP outcomes are better than medication, the nocturia response remains unsatisfactory after TURP.

Several studies have demonstrated that MetS and its components are associated with LUTS. Cosimo et al. conducted a retrospective study that showed that age, MetS, PV, and smoking were independent risk factors for the severity of nocturia (10). In addition, a separate study showed that PV, MetS, and smoking were associated with moderate/severe persistent nocturia after TURP in multivariate analysis (8). Regrettably, this study only described risk factors for the severity of postoperative nocturia. Considering the effect of preoperative nocturnal voids, we investigated the risk factors for unsatisfactory improvement (<50% reduction) in nocturia after HoLEP. In contrast to their findings, we found that age was associated with unsatisfactory improvement in nocturia after surgery.

Although the mechanism is not yet fully understood, several works have reported a positive correlation between obesity and nocturia. The Boston Area Community Health (BACH) survey reported that BMI > 30 increases the risk of nocturia (OR = 1.65, 95% CI: 1.29–2.11) (20). Similarly, other studies (21, 22) also reported that obesity is significantly associated with nocturia. Abdominal obesity may increase intra-abdominal pressure, leading to a reduction in bladder capacity, to consequent increase in nocturia (23). Additionally, a randomized clinical trial study reported an association between weight loss and improvement in nocturia, with a much lower incidence of nocturia in behavioral weight loss than in the control group over a 6-month period (24). In our study, a significant association between obesity and improvement in nocturia has not been reported.

Studies have reported that type 2 diabetes is closely related to nocturia (20, 25–27). Therefore, osmotic diuresis should not be ignored. Patients with diabetes have a higher incidence of nocturia than those without (27). Similarly, Fitzgerald et al. (20) reported a 1.67-fold increase in the risk of nocturia in patients with diabetes. Consistent with previous studies, our results showed that diabetes also affected nocturia improvement after HoLEP. An animal study (28) has shown that diabetes may affect bladder uroepithelial homeostasis and further contribute to bladder dysfunction. Hyperinsulinemia may lead to the activation of the sympathetic nervous system, and may be associated with increased prostatic smooth tone, leading to more severe LUTS (29).

In a Japanese study that included 728 patients with LUTS, multivariate analysis revealed that hypertension was significantly associated with nocturia (OR = 9.79, 95% CI: 6.53–14.9) (30). Moreover, blood pressure is significantly higher in elderly patients with nocturia than in those without (31). Hwang et al. reported that PV was relatively large in patients with poorly controlled blood pressure, which leads to increased functional bladder residual urine and consequently more nocturia (32). Additionally, sodium retention is an important mechanism leading to nocturnal polyuria in patients with hypertension, resulting in increased urine production.

However, a less clear association was observed between dyslipidemia and LUTS/nocturia. An overactive bladder and prostate enlargement, resulting in increased nocturnal voids, have been shown to occur in hyperlipidemic rats (33). A significant association between hypertriglyceridemia and nocturia (≥2 voids) was reported in a population-based epidemiological survey (34). However, an association between nocturia and dyslipidemia has not been found in other epidemiological studies (35).

It is well known that the prevalence of nocturia increases with age (3). In our study, age was an independent risk factor for unsatisfactory nocturia improvement after HoLEP. It is easy to understand that older men are more likely to develop bladder outlet obstruction, resulting in decreased functional capacity of bladder including impaired contraction strength and reduced storage function. Reduced bladder capacity (36) and overactive bladder (37) are also more common in the elderly. Older patients may have more comorbidities such as hypertension, diabetes and obesity, which contribute to nocturia. Therefore, eliminating the bladder outlet obstruction in these patients is an important intervention to reduce nocturia, but it alone is not sufficient.

This study had some limitations. First, there may have a selection bias because it was a retrospective study. This was a single-center study with a small sample size, which is less representative of the Chinese population. Second, considering the 24-h water intake, urine amount and nocturnal bladder capacity, the lack of frequency volume charts to evaluate nocturia is an important limitation of the present study. As in other studies (8, 10), we evaluated nocturia episodes using IPSS item 7, which may be inaccurate in the assessment of patients with more than five preoperative nocturia episodes. Moreover, the postoperative follow-up period was only 3 months, without long-term follow-up. Finally, we conducted this study in a Chinese patient cohort, and the findings may not be applicable to European or American populations.

In conclusion, our results suggest that age and MetS are significantly associated with unsatisfactory improvement in nocturia (<50% reduction) after HoLEP in the Chinese patients with BPH. It would be interesting for future studies to evaluate the effect of lifestyle on the improvement of nocturia after HoLEP in patients with BPH. Although the mechanism is not fully understood, counseling BPH patients with MetS about postoperative nocturia improvement is warranted.

## Data Availability

The original contributions presented in the study are included in the article/supplementary material, further inquiries can be directed to the corresponding author/s.
